# STEP EXECUTION TIME: EXAMINING ITS RELIABILITY AND ASSOCIATION WITH PARTICIPATION IN OLDER ADULTS

**DOI:** 10.1093/geroni/igac059.2320

**Published:** 2022-12-20

**Authors:** Chad Tiernan, Ida Kolodziejczyk, Susan Talley, Allon Goldberg

**Affiliations:** University of Michigan-Flint, Flint, Michigan, United States; University of Michigan-Flint, Flint, Michigan, United States; University of Michigan-Flint, Ferndale, Michigan, United States; University of Michigan-Flint, Flint, Michigan, United States

## Abstract

The projected rise in the number of older adults warrants increased attention to selecting reliable and meaningful clinical assessments. Compared to more frequently used balance measures, step execution time (SET) has not been studied extensively. The purpose of the current study was twofold. First, we investigated the association of several balance measures [SET, Activities-specific Balance Confidence Scale (ABC-6), Five-times-sit-to-stand (5TSTS), Four-square step test (4SST), and maximum step length (MSL)] with participation (LIFE-H). Second, reliability of SET was investigated. The study included 32 community-dwelling older adults between the ages of 65 and 83 years (88% White, 66% female). Results indicated that SET was the only balance measure associated with both participation accomplishment (r = -.54, p = .001) and participation satisfaction (r = -.55, p = .001); 5TSTS (r = -.64, p < .001) and 4SST (r = .37, p = .039) were also significantly correlated with participation accomplishment while MSL (r = .37, p = .040) showed a significant association with participation satisfaction. SET demonstrated excellent test-retest reliability (ICC = .92). Bland-Altman analysis determined the 95% Limits of Agreement to be -258.5 ms to +271.5 ms (mean difference = 6.5 ms; 95% CI of difference = -43.1 to 56.1), suggesting that bias was not a concern. SEM (100.5 ms) and MDC95 (278.5 ms) for SET represented 9.3% and 25.8% of the mean, respectively. Collectively, findings suggest that SET may have clinical utility as a reliable assessment in older adults that relates to meaningful constructs, such as participation.

